# Supplementary material for: Genomic Landscape Alterations in Primary Tumor and Matched Lymph Node Metastasis in Hormone-Naïve Prostate Cancer Patients
Source: Cancers (Basel). 2022 Aug 30;14(17):4212. doi: 10.3390/cancers14174212 (PMC9454441; doi:10.3390/cancers14174212)

Supplementary File

Materials and Methods

Prospectus

Eleven patients out of 17 were affected by prostate cancer with metastatic lymph nodes, so three samples were extracted for each of these patients (adjacent normal prostate tissue samples, tumor and lymph node). The remaining six patients developed a tumor of a non-metastatic nature of lymph nodes, so we extracted just a sample of adjacent normal prostate tissue samples and a sample of the primary tumor. All of these samples, 45 in total, underwent paraffinization for storage and transport pending DNA extraction.

Deparaffinization Procedure and DNA Extraction

The paraffin drums containing the samples were initially treated in such a way as to eliminate any excess of pure paraffin. DNA was extracted from the tissue obtained with deparaffinization procedure using the same kit according to the manufacturer's instructions. DNA was quantified using the fluorimeter Qubit dsDNA BR Assay Kit (Ref. 32850, Invitrogen, 92008 Carlsbad, CA, USA).

Table S1. Number of patients, type of sample and quantification of all 45 samples.

Patient	***Sample***		***DNA Quantification (ng/µL)***	***Patient***	***Sample***		***DNA Quantification (ng/µL)***
	**CH1**	Tumor	201		**CH22**	Tumor	67,6
**1**	**CH2**	Adjacent normal prostate tissue	200	**8**	**CH23**	Adjacent normal prostate tissue	71
	**CH3**	Lymph node	5,49		**CH24**	Lymph node	65,5
	**CH4**	Tumor	345		**CH25**	Tumor	22,4
**2**	**CH5**	Adjacent normal prostate tissue	138	**9**	**CH26**	Adjacent normal prostate tissue	58,8
	**CH6**	Lymph node	160		**CH27**	Lymph node	73,2
	**CH7**	Tumor	149		**CH28**	Tumor	122
**3**	**CH8**	Adjacent normal prostate tissue	105	**10**	**CH29**	Adjacent normal prostate tissue	84,2
	**CH9**	Lymph node	236		**CH30**	Lymph node	140,6
	**CH10**	Tumor	44		**CH31**	Tumor	51
**4**	**CH11**	Adjacent normal prostate tissue	27,6	**11**	**CH32**	Adjacent normal prostate tissue	82
	**CH12**	Lymph node	0,231		**CH33**	Lymph node	116
	**CH13**	Tumor	48,5		**CH34**	Tumor	93,6
**5**	**CH14**	Adjacent normal prostate tissue	25,9	**12**	**CH35**	Adjacent normal prostate tissue	24,2
	**CH15**	Lymph node	39,6	**13**	**CH36**	Tumor	92,4

	**CH16**	Tumor	46,8		**CH37**	Adjacent normal prostate tissue	26,6
**6**	**CH17**	Adjacent normal prostate tissue	41,7	**14**	**CH38**	Tumor	102
	**CH18**	Lymph node	47,5		**CH39**	Adjacent normal prostate tissue	95,4
	**CH19**	Tumor	74,2	**15**	**CH40**	Tumor	76
**7**	**CH20**	Adjacent normal prostate tissue	37,6		**CH41**	Adjacent normal prostate tissue	70,8
	**CH21**	Lymph node	84,2	**16**	**CH42**	Tumor	99,6
					**CH43**	Adjacent normal prostate tissue	54,8
				**17**	**CH44**	Tumor	89,2
					**CH45**	Adjacent normal prostate tissue	99,2

Quality control

The samples that underwent this quality control were CH3, CH10, CH11, CH12, CH13, CH14, CH15, CH18, CH30, CH38, CH39, CH42 and CH43. The CH12 sample had a too-low DNA concentration, so it was not possible to sequence it a second time, since the whole volume was used for the first sequencing.

Table S2. Threshold cycle resulting from RT-PCR for Illumina® FFPE QC Kit. Acceptable values are ΔCTs ≤ 2,00.

Sample	*Threshold Cycle*	*Average CT*	*ΔCTs*	*Sample*	*Threshold Cycle*	*Average CT*	*ΔCTs*
**QCT**	16,93<br>17,88<br>17,65	17,49	0,00	**CH18**	18,89<br>18,85<br>18,78	18,84	1,35
**CH3**	17,22<br>17,64<br>17,18	17,35	-0,14	**CH30**	17,20<br>17,52<br>17,25	17,32	-0,16
**CH10**	19,73<br>19,38<br>19,20	19,44	1,95	**CH38**	18,73<br>18,84<br>18,70	18,76	1,27
**CH11**	20,28<br>19,94<br>19,96	20,06	**2,57**	**CH39**	18,73<br>18,56<br>18,51	18,60	1,11
**CH13**	18,50<br>18,75<br>18,67	18,64	1,15	**CH42**	20,39<br>20,56<br>20,23	20,39	**2,91**
**CH14**	19,21<br>18,87<br>19,19	19,09	1,60	**CH43**	20,31<br>20,33<br>20,29	20,31	**2,82**
**CH15**	19,01<br>18,93<br>19,37	19,10	1,62	**NTC**	36,73<br>36,28<br>30,98	34,66	17,18

Results

DNA Amplicon Sequencing

Table S3. (a) Average quality data for all samples.

Total PF Reads	Percent Q30 Bases	Percent-On target Aligned Reads	Autosome Call rate	
931.376	97.77%	98.51%	99.95%	
		**Read Level Statistics**		
**Read**	**Total Aligned Reads**	**Percent Aligned Reads**		
1	389.747	82,31%		
2	386.019	82,91%		
		**Base Level Statistics**		
**Read**	**Percent Q30 Bases**	**Total Aligned Bases**	**Percent Aligned Bases**	**Percent Mismatches**
1	98.36%	39.863.517	71,15%	0,24%
2	96.98%	39.468.634	70,55%	0,28%
**Coverage Summary**				
**Amplicon Mean Coverage**		**Uniformity of Coverage (Pct >0.2 mean)**		
3689,9		98.12%		

Table S3. (b) MGS percentage of all patients.

	Percentage		
	Tropism	MAF	ClinVar
1	10.0	60.0	30.0
2	17.8	50.4	31.8
3	8.6	65.7	25.7
4	23.4	51.1	25.5
5	32.6	41.5	25.9
6	33.3	44.4	22.2
7	26.5	45.5	28.0
8	28.5	49.7	21.8
9	28.8	45.5	25.8
10	33.7	48.1	18.3
11	23.3	51.2	25.6
12	6.7	68.9	24.4
13	7.7	69.2	23.1
14	20.3	50.8	28.8
15	12.9	48.4	38.7
16	16.7	55.1	28.2
17	15.9	58.0	26.1
**Average**	**20.4**	**53.1**	**26.5**

Table S4. Sequenced mutations distribution in all samples.

	Mucosa			***Tumor***			***Lymph Node***		
	Intronic	Synonymous	Nonsynonymous	Intronic	Synonymous	Nonsynonymous	Intronic	Synonymous	Nonsynonymous
ABL1	0	1	1	0	1	1	0	3	1
AKT1	0	0	0	0	0	1	0	0	0
APC	0	7	0	0	8	2	0	6	1
ATM	0	0	1	1	0	1	0	0	2
CDH1	0	0	1	0	0	1	0	0	0
CSF1R	0	0	16	0	0	15	0	0	9
CTNNB1	0	0	0	0	0	2	0	0	2
EGFR	0	4	5	0	4	5	0	3	4
ERBB2	0	0	0	0	1	1	0	0	0
ERBB4	19	0	6	20	0	14	15	0	9
FGFR2	0	0	0	0	0	1	0	0	0
FGFR3	0	13	1	0	13	2	0	10	1
FLT3	15	0	13	15	0	16	11	0	10
HNF1A	0	0	0	0	1	0	0	0	0
HRAS	0	6	0	0	6	1	0	5	1
IDH1	0	4	0	0	4	1	0	3	0
JAK3	0	0	3	0	0	3	0	0	3
KDR	12	1	9	12	1	9	9	1	5
KIT	0	2	3	1	2	3	0	2	2
MET	0	1	0	0	1	1	0	1	0
MLH1	0	0	0	0	0	1	0	0	0
MTTP	0	0	0	0	0	2	0	0	0
NPM1	12	2	0	12	2	0	8	2	1
PDGFRA	0	15	0	0	15	0	0	12	0
PIK3CA	4	3	6	4	3	7	4	3	7
RB1	0	0	0	0	0	0	2	0	1
RET	0	16	0	0	16	0	0	13	1
SMAD4	0	0	0	2	0	0	0	0	0
SMARCB1	4	0	2	4	1	2	4	0	3
STK11	2	0	1	2	0	2	2	0	0
TP53	0	0	16	0	0	17	0	0	9
VHL	0	1	1	0	2	1	0	1	1

Figure S1. Comprehensive analysis of ERBB4 expression profiles in prostate cancer

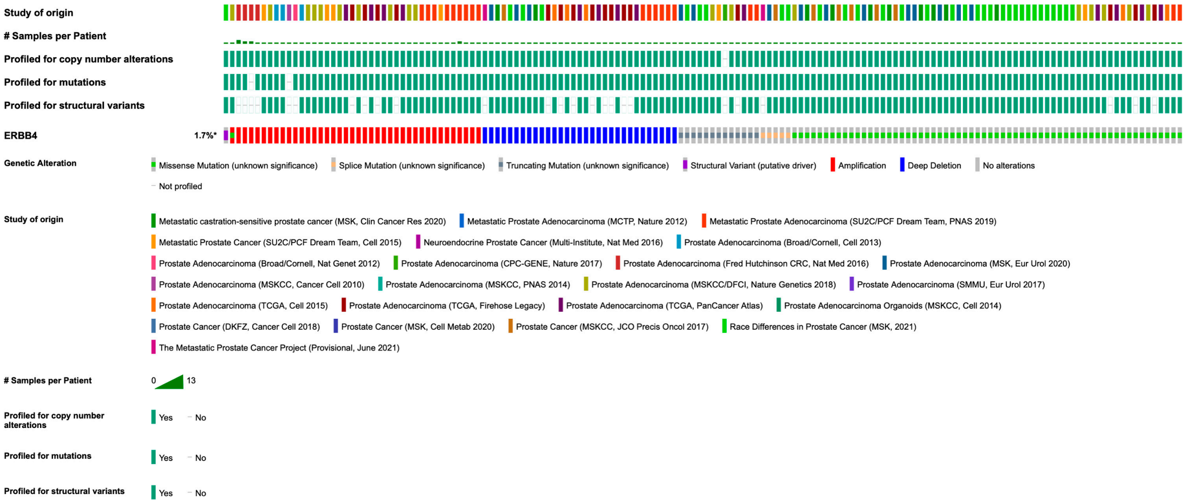

Figure S2. Comprehensive analysis of KIT expression profiles in prostate cancer

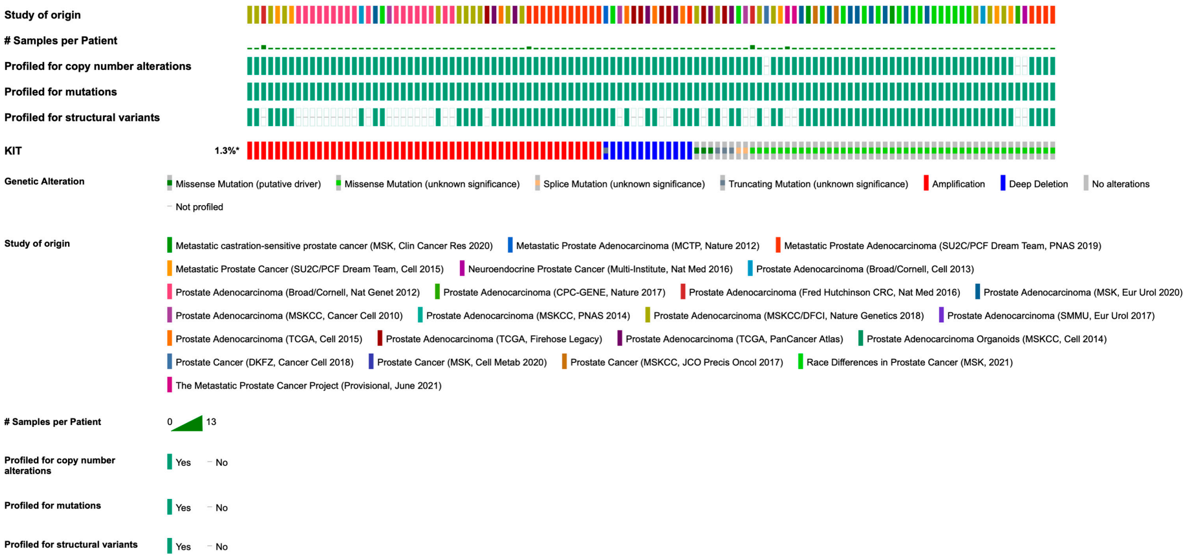

Figure S3. Comprehensive analysis of HRAS expression profiles in prostate cancer

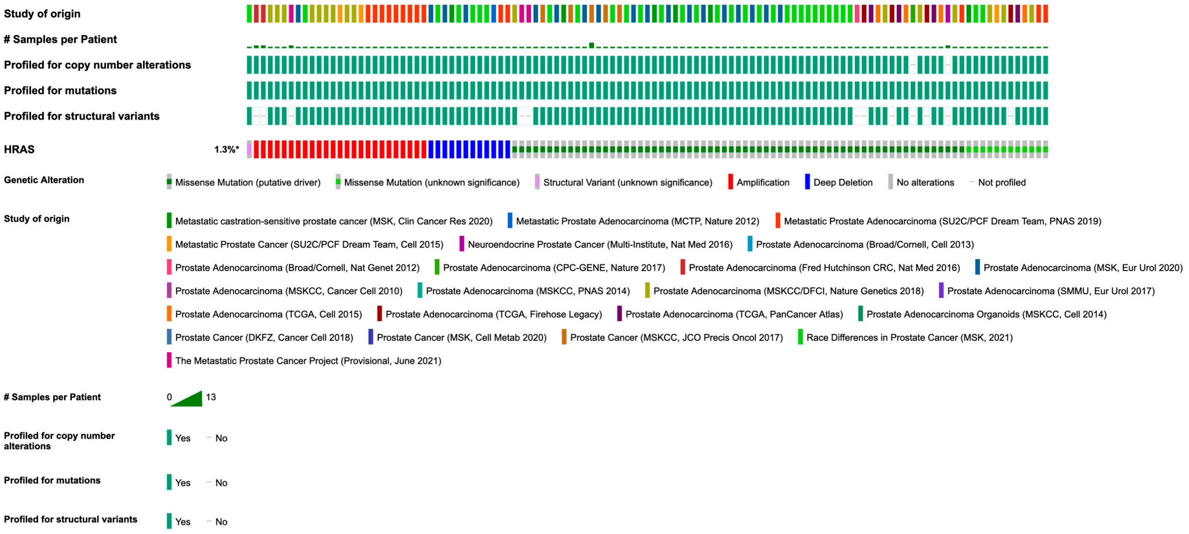

Figure S4. Comprehensive analysis of ALB1 expression profiles in prostate cancer

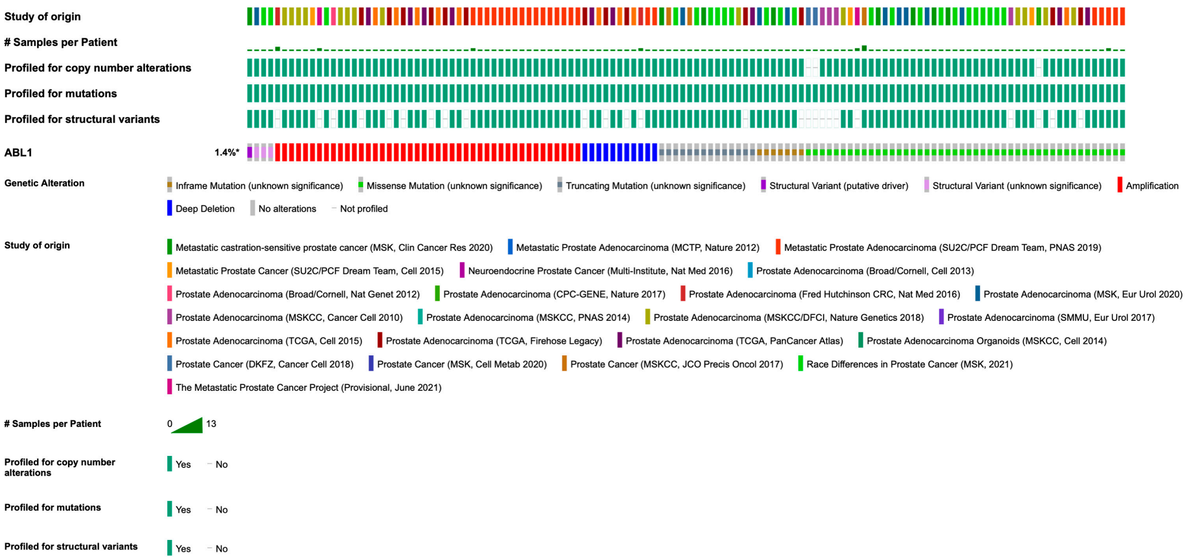

Figure S5. Comprehensive analysis of CTNNB1 expression profiles in prostate cancer

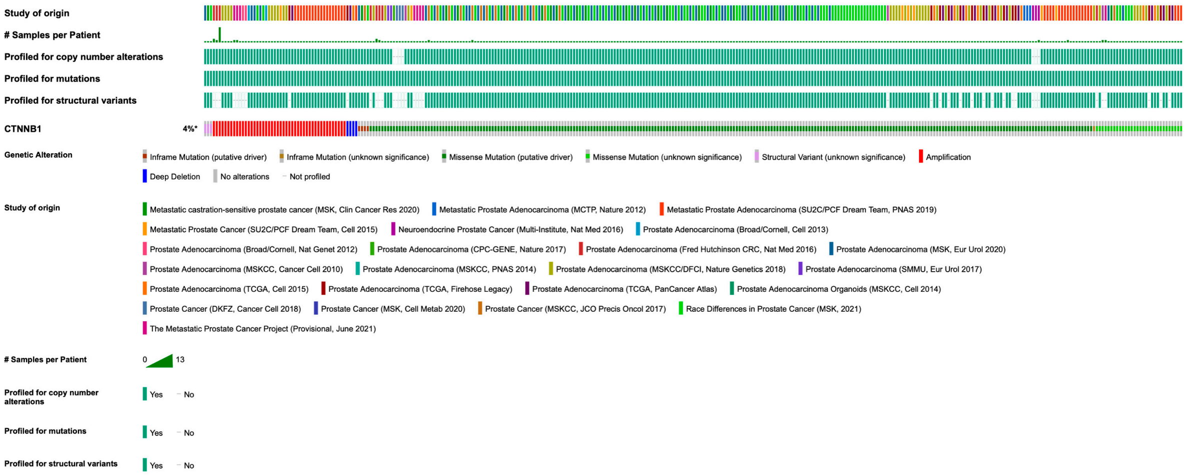

Figure S6. Expression of insulin receptor-alpha (A), insulin receptor-beta (B); insulin growth factor 1 receptor (C); androgen receptors (D) and PSMA (E) in prostate cancer

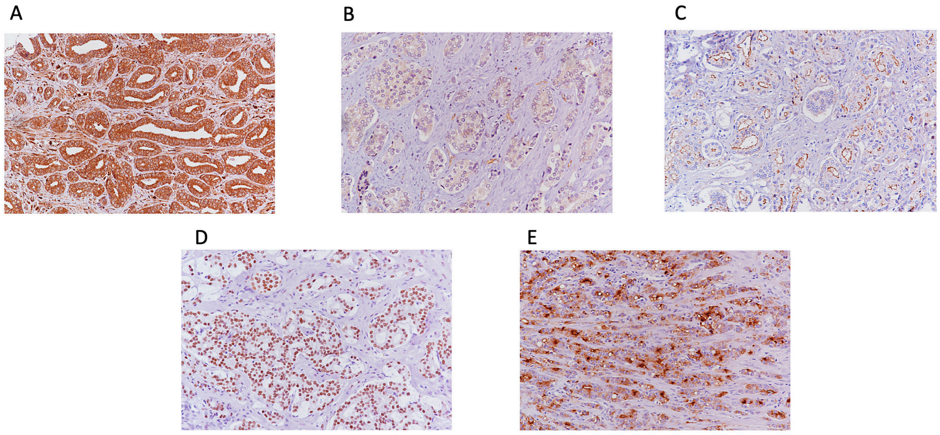

Supplement: Supplementary file 1 [file cancers-14-04212-s001.zip › cancers-1840952-supplementary.pdf]
